# The Gall Bladder

**Published:** 1911-09

**Authors:** J. L. Campbell

**Affiliations:** Professor of Surgical Anatomy and Clinical Surgery, Atlanta School of Medicine; Visiting Surgeon Wesley Memorial Hospital and Tabernacle Infirmary


### THE GALL BLADDER.

By J. L. Campbell, M. D.

*Professor of Surgical Anatomy and Clinical Surgery, Atlanta School of Medicine; Visiting Surgeon Wesley Memorial Hospital and Tabernacle Infirmary.*

This paper contemplates:

I. A brief study of the healthy gall bladder.

II. The etiology, symptomatology, complications and differental diagnosis of its sub-acute and chronic inflammatory diseases.

No consideration will be accorded the pathology of any form, nor will acute inflammations, perforation, empyema or gangrene be discussed.

I.

The gall bladder is a pear shaped musculo-membranous reservoir containing about 1½ oz. of bile, but capable of great distention. It has, according to W. J. Mayo, a two-fold function. *First,* by its power of distention it relieves the tension on the hepatic and common ducts during the active stage of liver secretion, thus preventing the pure bile being forced into the pancreas. *Second,* it secretes mucus which dilutes the bile so that if for any reason it is forced into the pancreas it is harmless, or excites only a mild pancreatitis.

The gall bladder is developed as a diverticulum from the common duct which originates as a secondary bud from the duodenum embracing the two primative liver buds which later become the right and left hepatic ducts. It is composed of the same embryonic tissue as the other portions of the foregut.

The gall bladder receives its blood supply from the cystic branch of the hepatic artery—a branch of the coeliac axis which also supplies the stomach, duodenum, pancreas, liver and spleen.

The nerve supply is largely sympathetic, coming from the great solar plexus through the coeliac and hepatic plexuses. There is a direct connection with the cerebro spinal centers and the gall-bladder through the right phrenic, the right great splanchnic and the 8th and 9th segments of the cord. The pneumogastrics also enter into the formation of the solar plexus and very probably send filaments to the gall-bladder. It is evident that the branch from the right phrenic which supplies the peritoneum of the diaphragm also supplies at least a part of the gall-bladder. Head has shown that when the gall-bladder is inflamed there is tenderness over the area of distribution of the right 8th and 9th Thoracic nerves and along the anterior border of the right trapezius muscle, thus proving the connection with the nerves that supply the skin of these areas.

The gall-bladder is poorly supplied with lymphatics, the walls having no glands and only a few channels. This accounts for the fact that fever and other constitutional symptoms are so rare where the body alone is involved.

II.

In order to give an opinion with reasonable certainty of confirmation at the operation we must study with care (A) the etiology, (B) the symptomatology and complications, and (C) differential diagnosis. As we are more likely to be confused by the sub-acute and chronic forms of the disease, I will confine my remarks largely to them.

(A) The etiology may be considered under (1) predisposing and (2) exciting causes.

(1) Of the predisposing causes we have—

(a) Age.
(b) Sex.
(c) Previous or existing illness.
(d) Habits.

(a) Cholecystitis and cholelithiasis occur far more frequently in persons of advanced age than in the young; probably because they are more subject to other predisposing causes. Seventy-five per cent. of cases are found in persons over 40; about one per cent. in persons under 20. A few cases have been reported in infants. (Osler's System of Medicine.)

Ochsner in Kelley & Noble's Gyn. & Abd. Surgery, quoting from Hartman says that the average age at which patients consult a surgeon in males of the laboring clases is 40, with a duration of symptoms for six years, of sedentary habits 37, duration of symptoms 9 years. In females of the laboring class 37½, duration of symptoms 7 years, of sedentary habits 37, with a duration of symptoms 9 years.

Moynheims found that the majority of patients consulted the physician between the ages of 40 and 45. W. J. Mayo in a recent article in the J. A. M. A. reported 41 cases under 20 years of age out of a series of 4,000 operated on at St, Mary's Hospital. 38 of these were females. The average age of my patients has been 44.8 years. One case, in which a large stone was removed from the common duct, was only 26 and had suffered for eight years with definite symptoms of common duct stones dating from her second pregnancy.

(b) Women are far more likely to have gall-bladder disease than men; 75 per cent. of the 4,000 cases operated on by the Mayos have been women. Ochsner says that 80 per cent. of his cases are women. Other authorities place the ratio at 4 to 1; 82 per cent. of my cases have been women.

Pregnancy is undoubtedly responsible for the great number of women having cholecystitis, for 90 per cent. of the married women in Mayo's cases had born children and 90 per cent. of these dated the beginning of their symptoms from a definite pregnancy. 80 per cent. of the women on whom I have operated have borne children.

(c) The history of previous or existing illness is also an important matter in considering the predisposing causes and is often of great benefit to us in arriving at a correct conclusion.

Appendicitis, typhoid fever, and intestinal indigestion or catarrh (so often caused by Cholecystitis that it is hard to say which precedes the other) deserve consideration in the order named.

Ochsner found that 35 per cent. of his cases had suffered with either acute or chronic appendicitis. MacCarthy found evidence of appendicitis in 13 per cent. in a pathological study of a series of 365 cases from the Mayo clinic. In my cases, 23.4 per cent. have had appendicitis and the appendix has been removed at the time of operation, except in one case where it had previously been removed without benefit. In addition to the above 21.5 per cent. of the women had pelvic disease as well as appendicitis making, in all, about 41 per cent. who had suffered with appendicittis.

There was a history of typhoid fever in 30 per cent. of my cases. Two of them less than one year. In one the gall-bladder symptoms seemed to begin with the convalescence and continued until the operation. The others had passed from one to several years before the onset of gall-bladder symptoms. In a series of 240 cases reported by J. B. Deavor 11.2 per cent. showed the presence of typhoid bacilli in cultures made from the bile, while 28.3 per cent. of the same series showed colon bacilli in the cultures.

It is highly probable that the ptosis usually accompanying intestinal catarrh may bear some relation to the cause of cholecystitis as it prevents free drainage of the gall-bladder. It is a well known fact that where there is residual bile it forms a splendid culture medium. Adhesions of the gall-bladder to the neighboring viscera—especally to the duodenum, organic heart and kidney disease, pneumonia and many other conditions, by lowering the body resistance, bring about changes which favor gall-stone formations.

(d) Tight lacing, sedentary and indoor habits tend to constipation, thus filling the intestine with bacteria which naturally gives the liver more work to do and lessens the bacteriacidal [illegible] of the bile.

(2) Infection is always the existing cause but cannot become effective so long as some of the predisposing causes do not exist.

The infection may reach the gall-bladder (a) by means of the portal circulation, (b) by the systemic circulation, (c) the lymphatics, (d) extension along the common duct and (e) extension from the general peritoneal cavity through the walls of the gall-bladder. It is probable that these take place in the order named.

(B) The symptoms of cholecystitis are exceedingly variable, patients with marked pathological changes, many stones in the gall-bladder and even a stone in the common duct, may have periods of apparently perfect health. while others with only a catarrhrl inflammation and a few adhesions suffer great inconvenience.

Stomach symptoms are probably the most constant. There is a sense of weight and fullness about the epigastrium often relieved by eructations of gas. There is a burning sensation in the stomach accompanied by great nervousness; indigestion is nearly always present, but the symptoms have no regular periodicity with reference to food. Nausea is an almost constant symptom during the attack of "colic," and relief frequently follows a complete emptying of the stomach either by vomiting or lavage.

The irritation within the gall-bladder disturbs the function of the stomach because, as we have tried to show, the two are closely related embryologically, anatomically and physiologically.

Pain is a variable symptom so far as the severity is concerned. Nearly all cases suffer some pain, but by no means so large a number as was formerly believed. During an attack of colic the pain is very severe, cramping and lancinating, and the patient calls loudly for relief. This pain is usually felt just to the right of the median line radiating over the upper right quadrant of the adbomen and may be felt beneath the sternum, finally to concentrate around the gall-bladder. When this symptom is present there is little danger of making a mistake in the diagnosis. If the symptoms are of a sub-acute or chronic continuous type, as so often occurs, we experience more difficulty.

In a majority of such cases there will be an indefinite history of pain and tenderness over the distribution of the posterior division of the right 8th and 9th thoracic nerves and sometimes where the lateral branches are given off. A few patients will have a tender point about two inches above the right shoulder tip along the anterior border of the trapezius muscle. These points are sometimes unnoticed by the patient but can be easily demonstrated by pressure. Occasionally there is a sense of constriction as if something was tied around the body just above the waist.

The skin may be hyperasthetic over the area of distribution of the 8th and 9th thoracic nerves. Pain about the fundus of the gall-bladder may be experienced from a change of position, as turning from side to side, or arising from a sitting posture.

Murphy has emphasized the fact that if we press with the tips of our fingers over the fundus of the gall-bladder and ask the patient to cough there will be such a sudden contraction of the diaphragm when the fundus is brought into contact with the depressed abdominal wall that the cough will be arrested. There is frequently rigidity of the right rectus muscles; pressure over the appendix will sometimes cause pain in the gall-bladder area.

In my cases 41.1 per cent. have had acute attacks of colic with periods of good health except for indigestion. Certain articles of food will often precipitate an attack of colic. Tenderness over the gall-bladder was not always present in the interval. 29.4 per cent. gave a history of one more or less severe attack with the symptoms continuing, but not severe enough to confine them to bed or prevent them doing a certain amount of work. There was in these cases a tender spot below the right scapular and one of them had tenderness and hyperasthesia over the area of distribution of the right eighth and and ninth thoracic nerves.

Jaundice is by no means a constant symptom, and when present means a complication. Perhaps the most frequent cause of jaundice is (a) stone in the common duct. Jaundice from this cause is usually intermittent in character, due to the "ball valve" condition produced by dilatation of the common uct behind the obstruction, which when relieved by the regurgitation of the stone into the dilated pouch allows the bile to flow in the normal course, and the jaundice clears up. This condition may also be accompanied by chills and fever and is frequently mistaken for malaria.

(b) Jaundice due to a growth, benign or malignant of or near the common duct, or of the liver, is of a constant and increasing character, growing worse from day to day.

(c) Cholangitis may be caused by the passage of a stone or plug of infected mucous through the common duct and a jaundice result which will generally clear up in a few days.

(d) The jaundice dependent on pancreatitis will be more or less severe according to the involvement of the pancreas which may be due to plugging of the cystic duct and pure bile being forced into the pancreas; 23.5 per cent. of my cases have had jaundice and I have invariably found one of the above complications.

(C) The Mayos have found it most difficult to differentiate between peptic ulcer and cholecystitis. In peptic ulcer there is more regularity to the periodicity and more definite relation to the intake of food. The pain is not so severe and differs much in character. In the majority of cases of peptic ulcer there is likely to be blood in the vomitus or in the stools, though in severe cases of gall-stone colic, blood may be vomited—due to the strain, sour stomach or "heart burn" is more pronounced in ulcer than cholecystitis.

Chronic pancreatitis may be mistaken for cholecystitis but if a careful examination of the stools is made after a series of test meals the diagnosis can usually be cleared up.

Appendicitis may be mistaken for cholecystitis. In one of my cases the appendix had been removed without benefit to the patient.

It must be remembered that a perforating ulcer of the duodenum may be mistaken for gall-stone colic. The condition can only be detected by careful and repeated examinations of the blood.

834Candler Bldg.

